# 

**Published:** 1885-06

**Authors:** 


					﻿lo-Cts. a copy.
JUNE, 1885.
Ii.oo per year.
HAL£5«
gJoVRNALl
HEALTH
ESTABLISHED 1854
Vi-3 2
3^ 6
OFFICE, 75 & 77 BARCLAY STREET, NEW YORK.
Eutered as Second-clasH Matter a' the Post-Office, New York.
				

